# Complete mitochondrial genome of the parrotfish *Calotomus japonicus* (Osteichthyes: Scaridae) with implications based on the phylogenetic position

**DOI:** 10.1080/23802359.2016.1214553

**Published:** 2016-09-04

**Authors:** Kohji Mabuchi

**Affiliations:** Atmosphere and Ocean Research Institute, The University of Tokyo, Kashiwa, Chiba, Japan

**Keywords:** Labriformes, mitogenome, cytb, *Scarus ovifrons*

## Abstract

The complete mitochondrial genome sequence was determined for a specimen of *Calotomus japonicus*, a temperate parrotfish endemic to coastal East Asia. It was compared phylogenetically with previously published partial sequences from this species and other parrotfishes. The obtained tree indicated that the three cytb sequences of *C. japonicus* from a recent molecular study (LC068806-8) probably resulted from introgression through intergeneric hybridization, or possibly from sample confusion. Taking the presently obtained mitogenome as representative of *C. japonicus*, the species most closely related to this one among congeners is *C. zonarchus*, which is endemic to the Hawaiian islands.

*Calotomus japonicus*, which belongs to the family Scaridae, is a temperate parrotfish endemic to East Asian coastal waters. Within this mainly tropical family, only this species and *Scarus ovifrons* are common in the waters of southern Japan (Kishimoto 1988). Although the consumption of *S. ovifrons* sometimes causes poisoning in humans (Suzuki et al. 2013), *C. japonicus* is an important target species for local fisheries (Kume et al. 2010). These two species belong to different genera and are easily distinguished by their morphological features (Shimada 2013). A recent molecular study by Ogawa et al. (2015), however, reported that 477-base-pair (bp) fragments of the mitochondrially encoded cytochrome b (cytb) gene were almost identical between them, and further that the cytb haplotypes from *C. japonicus* were clustered not into the clade of *Calotomus*, but into that of *Scarus*. Considering their taxonomic status, I suspected that the cytb sequences of *C. japonicus* reported by the authors might not be representative of this species. To investigate this possibility, I determined the complete mitochondrial DNA sequence of *C. japonicus*, and compared it phylogenetically with the cytb sequences reported by Ogawa et al. (2015) and other previously published sequences of parrotfishes.

The complete mitochondrial genome (mitogenome) of *C. japonicus* was determined by a polymerase chain reaction (PCR)-based technique (Mabuchi et al. 2004) using a specimen purchased at Odawara Fish Market in Kanagawa Prefecture, Japan, and deposited at the National Science Museum, Tokyo (registered number NSMT-P 65394). The obtained mitogenome (DDBJ accession no. AP017568) is 17,199 bp in length, which is *ca*. 500–520 bp longer than the five parrotfish mitogenomes reported thus far (Table 1). Most of the differences in length result from variation in the length of intergenic regions: the control region of *C. japonicus* is longer than those of the five parrotfishes by *ca*. 400 bp, and the intergenic region between tRNA^Ser^ and tRNA^Leu^ of the species is *ca*. 30 bp longer than those of the others (Table 1). As already reported by Mabuchi et al. (2004), this species shares a gene rearrangement accompanied by a tRNA pseudogene with other parrotfishes. In the typical gene order of vertebrates, the tRNA gene cluster between the ND1 and ND2 genes includes the tRNA^Ile^ (I), tRNA^Gln^ (Q), and tRNA^Met^ (M) genes in this order (IQM). However, in the parrotfish mitogenomes, the tRNA^Met^ gene is inserted between the tRNA^Ile^ and tRNA^Gln^ genes, and the tRNA^Gln^ gene is followed by a putative tRNA^Met^ pseudogene (Ψ_M_) (making the gene/pseudogene order IMQΨ_M_).

**Table 1.** Sequence lengths (base pairs) of whole and major intergenic regions of six parrotfish mitogenomes.

	Regions				
	Wholemitogenome	Between tRNA^Pro^ and tRNA^Phe^(CR^a^)	Between tRNA^Gln^and ND2(Ψ_M_^b^)	Between tRNA^Asn^ and tRNA^Cys^(O_L_^c^)	Between tRNA^Ser^and tRNA^Leu^
*Calotomus japonicus* AP017568	17,199	1,313	70	39	39
*Chlorurus sordidus* AP006567	16,681	920	67	44	10
*Scarus forsteni* NC_011928	16,679	919	66	41	11
*Scarus ghobban* NC_011599	16,676	916	66	42	11
*Scarus rubroviolaceus* NC_011343	16,681	921	66	41	11
*Scarus schlegeli* NC_011936	16,701	920	66	39	9

^a^Control region.

^b^tRNA^Met^ pseudogene.

^c^Origin of light-strand replication.

Using the six mitogenomes and other available partial sequences of parrotfishes, a phylogenetic tree was constructed using a supermatrix approach (de Queiroz & Gatesy 2007) by the method described in the legend of Figure 1. The resultant supermatrix tree recovered the two major parrotfish clades of Streelman et al. (2002): one was the “Seagrass” clade including *Cryptotomus*, *Nicholsina*, and *Sparisoma*, as well as *Calotomus*, and the other was the “Reef” clade including *Scarus* and *Chlorurus,* although the phylogenetic position of *Leptoscarus* differed from that in the previous study, in which this genus was included in the “Seagrass” clade. The presently obtained mitogenome of *C. japonicus* was nested within the “Seagrass” clade, and formed a *Calotomus* clade together with a partial 12S rRNA sequence of this species and the cytb and COI sequences of the other congeneric species. The cytb sequences of *C. japonicus* reported by Ogawa et al. (2015) (indicated by arrows) were, on the other hand, included in the “Reef” clade, and showed a very close relationship with the cytb sequences of *S. ovifrons*, as shown in Supplementary Material 1 in the report by Ogawa et al. (2015). Based on these results, I concluded that the presently obtained mitogenome of *C. japonicus* is more representative of this species than the cytb sequences of *C. japonicus* reported by Ogawa et al. (2015). The latter sequences (LC068806-8) probably resulted from introgression through intergeneric hybridization between *C. japonicus* and *S. ovifrons*, or possibly from sample confusion.

**Figure 1.**
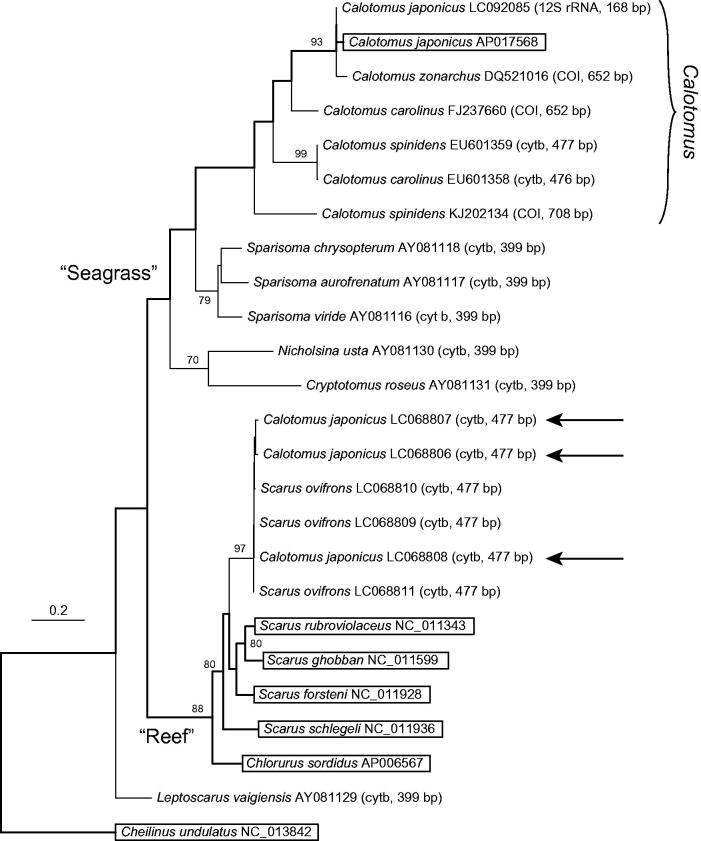
Supermatrix tree of 24 selected mitochondrial DNA sequences of parrotfishes with a labrid fish, *Cheilinus undulatus*, as an outgroup. Accession numbers are indicated after the species names with gene regions and their lengths in parentheses for partial sequences. Bootstrap support (70% or over) is indicated at the nodes. For the three arrows, see the text. The tree backbone indicated by bold lines was first generated for the boxed species (for which mitogenome sequences were available) by partitioned maximum-likelihood (ML) analysis using 13 protein-coding and two ribosomal RNA gene sequences, as follows. Multiple alignment was conducted for each of the 15 genes using the online version of MAFFT (http://mafft.cbrc.jp/alignment/server/), and ambiguous regions were trimmed using trimAl (ver. 1.2; Capella-Gutiérrez et al. 2009) with the “strict” setting. For the concatenated 13,751-bp dataset, the optimal partition model was determined using PartitionFinder (ver. 1.1.1; Lanfear et al. 2012) with codons of each protein-coding gene considered separately. Using the selected model including 10 partition subsets, ML analysis was performed using RAxML (ver. 8.1.5; Stamatakis 2014) with the following settings: -f a; -m GTRGAMMA; -# 1000. The obtained ML tree was then used as a backbone constraint for the supermatrix tree. The supermatrix tree was constructed based on the dataset including the 25 sequences, which were prepared by the same methods as for the backbone tree. For the resultant 13,758-bp dataset, ML analysis was performed by RAxML using the -r option and the same partition model as for the backbone tree, with the other settings as follows: -f a; -m GTRCAT; -# 1000; -V.

The presently obtained mitogenome of *C. japonicus* revealed an interesting biogeographic pattern within the genus *Calotomus*. This genus consists of five species (Randall 1985), and the present dataset included four of the five (only *Calotomus viridescens*, endemic to the Red Sea, was absent). Based on the present supermatrix tree (Figure 1), the species most closely related to *C*. *japonicus*, which is endemic to coastal East Asia, is neither *C. carolinus* nor *C. spinidens*, both of which are widely distributed in the Indo-West Pacific region, but a species geographically isolated from *C. japonicus*, *C. zonarchus*, endemic to the Hawaiian islands.
